# Learning regularized representations of categorically labelled surface EMG enables simultaneous and proportional myoelectric control

**DOI:** 10.1186/s12984-021-00832-4

**Published:** 2021-02-15

**Authors:** Alexander E. Olsson, Nebojša Malešević, Anders Björkman, Christian Antfolk

**Affiliations:** 1 Department of Biomedical Engineering, Faculty of Engineering, Lund University, Lund, Sweden; 2 Department of Hand Surgery, Institute of Clinical Sciences, Sahlgrenska Academy, Sahlgrenska University Hospital and University of Gothenburg, Gothenburg, Sweden; 3 Wallenberg Center for Molecular Medicine, Lund University, Lund, Sweden

**Keywords:** Electromyography, Prosthetic control, Online performance, Regression, Deep learning, Representation learning, Regularization, Multitask learning

## Abstract

**Background:**

Processing the surface electromyogram (sEMG) to decode movement intent is a promising approach for natural control of upper extremity prostheses. To this end, this paper introduces and evaluates a new framework which allows for simultaneous and proportional myoelectric control over multiple degrees of freedom (DoFs) in real-time. The framework uses multitask neural networks and domain-informed regularization in order to automatically find nonlinear mappings from the forearm sEMG envelope to multivariate and continuous encodings of concurrent hand- and wrist kinematics, despite only requiring categorical movement instruction stimuli signals for calibration.

**Methods:**

Forearm sEMG with 8 channels was collected from healthy human subjects (N = 20) and used to calibrate two myoelectric control interfaces, each with two output DoFs. The interfaces were built from (I) the proposed framework, termed *Myoelectric Representation Learning* (MRL), and, to allow for comparisons, from (II) a standard pattern recognition framework based on Linear Discriminant Analysis (LDA). The online performances of both interfaces were assessed with a Fitts’s law type test generating 5 quantitative performance metrics. The temporal stabilities of the interfaces were evaluated by conducting identical tests without recalibration 7 days after the initial experiment session.

**Results:**

Metric-wise two-way repeated measures ANOVA with factors method (MRL vs LDA) and session (day 1 vs day 7) revealed a significant ($p<0.05$) advantage for MRL over LDA in 5 out of 5 performance metrics, with metric-wise effect sizes (Cohen’s $d$) separating MRL from LDA ranging from $\left|d\right|=0.62$ to $\left|d\right|=1.13$. No significant effect on any metric was detected for neither session nor interaction between method and session, indicating that none of the methods deteriorated significantly in control efficacy during one week of intermission.

**Conclusions:**

The results suggest that MRL is able to successfully generate stable mappings from EMG to kinematics, thereby enabling myoelectric control with real-time performance superior to that of the current commercial standard for pattern recognition (as represented by LDA). It is thus postulated that the presented MRL approach can be of practical utility for muscle-computer interfaces.

## Background

Muscle-computer interfaces (MCIs) have found use in a broad range of clinical and biotechnical domains [1]. Most salient within the category of clinical applications is perhaps the field of hand- and wrist prosthetics, where myoelectrically controlled prostheses have been part of clinical routine since the 1960s [2]. In this application, electromyography (EMG) signals are processed by an MCI and transformed into movement commands intended to modulate the behaviour of a powered actuator, i.e. a robotic replacement limb. The prototypical system [3] designed to this end utilizes a sparse setup of surface EMG (sEMG) electrodes which measure the activities of a single antagonistic muscle pair located superficially in the residual limb of the amputee. The difference in some measure of intensity (e.g. signal magnitude) between the sEMG signals from the pair can thereafter be mapped directly to the force driving a single motorized degree of freedom (DoF) which is typically instantiated as the grasp aperture of a hand-replacing gripper. Within this framework, the additional DoFs possessed by multifunctional prostheses (which have recently become more available to hand- and arm amputees [4]) must be controlled sequentially by use of auxiliary protocols, e.g. based on co-contraction [5] or non-EMG inputs [6], for DoF switching. The enduring preponderance of this direct control framework can be understood in light of the robustness brought about by the relative simplicity of the relevant hard- and software, as well as the ease with which the intensity of contraction of a single muscle group can be controlled volitionally. However, disadvantages such as limited dexterity, lack of intuitiveness, and an associated cognitive burden have been observed among users [7]; these are thought to be among the main reasons for the high abandonment rates by which devices controlled in this way are afflicted [8].

The divide that separates the direct control paradigm from advances seen in mechatronics has for a time spurred research into potential alternatives. A noteworthy candidate to this end is the use of myoelectric pattern recognition [9–11]—a class of methods which formulates the control problem as one of supervised machine learning. Within this framework, example segments ${\varvec{X}}$ of a multichannel sEMG time series (typically acquired from the forearm) or more information-dense features [12] of the same, are, together with encodings of co-occurring movements ${\varvec{y}}$, fed to a machine learning algorithm which generates a computable function ${f}_{{\varvec{\theta}}}$. This learned function represents an approximate mapping between sEMG and movement and is typically derived by selecting the free parameters ${\varvec{\theta}}$ such that ${f}_{{\varvec{\theta}}}$ minimizes some loss metric $\sum_{t}\mathcal{L}({\mathbf{y}}_{{\varvec{t}}},{\widehat{{\varvec{y}}}}_{{\varvec{t}}},{\varvec{\uptheta}})$, where ${{\varvec{X}}}_{t}$ and ${{\varvec{y}}}_{t}$ are the sEMG segment and (a numeric encoding of) the concurrent movement, respectively, at time t, and ${\widehat{{\varvec{y}}}}_{t}={f}_{{\varvec{\theta}}}\left({{\varvec{X}}}_{t}\right)$ is (a numeric encoding of) the inferred movement. Following such initial calibration, ${f}_{{\varvec{\theta}}}$ can be used to process previously unseen segments by recognizing movement-specific sEMG patterns; an MCI based on pattern recognition can thus be understood as a form of gesture recognition system.

The contemporary engineering research literature shows no signs of scarcity when surveyed for approaches based on pattern recognition aiming to accommodate the mechanical sophistication of available robotic limbs. Algorithms from the broader machine learning discipline such as linear discriminant analysis [13]; support vector machines [14]; hidden Markov models [15]; and decision trees/random forests [16] have, among several others, been applied for this purpose; such methods have at times reached impressive classification accuracies of more than 95% for movement class sets with cardinalities exceeding 10 [17]. As in most other technical pursuits in which statistical inference plays a part, Deep Learning [18] in the form of, for example, convolutional neural networks (e.g. [19–23]) and recurrent neural networks (e.g. [24, 25]), has recently found widespread use in myoelectric control research [26] and has frequently attained exceptional accuracy scores. Unlike their ‘classical’ machine learning counterparts, such methods avert the need for manual feature engineering via their ability to gainfully operate directly on raw sEMG, but are often hampered by a need for time-consuming hyperparameter tuning; large datasets; and/or requirement on computational resources infeasible for embedded systems [27].

Independent of the minutiae of any specific algorithm, the improvements over the industrial and clinical status quo made possible by pattern recognition are quite apparent. Importantly, use of pattern recognition is congruent with complete naturalness of control: The task of mapping a detected movement attempt to a movement command corresponding to the very same movement is trivial, thus enabling an intuitive form of steering. Similarly, multiarticulate control can be realized either implicitly, by detecting separate multiarticulate movements and/or grasps as individual classes, or explicitly, by detecting each DoF separately using multi-output versions of pattern recognition [22, 28–30]. In spite of such alluring promises, the fact remains that remarkably few implementations of pattern recognition have so far been deployed at scale in the daily life of amputees [31].

Conjecturally, one of the main obstacles separating myoelectric pattern recognition from widespread adoption within prosthetics relates to the phenomenon of drift in the data-generating distribution $P({\varvec{X}}|{\varvec{y}})$ from which sEMG is sampled [32]. Stated succinctly, the statistical relationship connecting measured myoelectric activity ${\varvec{X}}$ to movement ${\varvec{y}}$ is not necessarily identical to the relationship which was valid at the time of calibration data acquisition, making the problem a specific instance of model overfitting. Variations in electrode positions; skin conductivity; limb placement and load; and fatigue are all examples of mechanisms which modulate the characteristics of the acquired sEMG [32], making the learned mapping ${f}_{\theta }({\varvec{X}})=\widehat{{\varvec{y}}}$ obsolete and thus degrading MCI performance over time [33]. Drift of this kind has in the past been mitigated either by including calibration data from a varied set of recording circumstances (although this approach has limitations regarding scalability [10]) or by using adaptive control strategies [34]. As will be argued in this paper, a complementary strategy is to develop methods which yield more generalizable mappings from sEMG to movement via regularization.

In addition to problems of robustness and stability of the aforementioned kind, one drawback of straightforwardly applying pattern recognition relates to proportionality of control. To make effective use of a prosthesis it is practical, and perhaps even necessary, to be able to not only transmit what movement to perform, but also to transmit information of the desired force and velocity—a capability not granted by basic pattern recognition. A naïve solution is to reformulate the classification problem as one of direct regression (of kinetics and/or kinematics), as is certainly notionally consistent [35]. However, at some point this requires ground truth measurements of relevant regressands, which in principle are impossible to acquire from prosthesis users. One way to circumvent this anatomical limitation has been the use of mirrored training [36], where sEMG from the amputation stump, collected during mediolaterally mirrored movements, is used to infer the kinematics of the contralateral, intact limb. Regression has also been realized by using continuous visual movement instruction stimuli as regressand [21], which requires the subject to manually vary the intensity of muscle contraction during acquisition of calibration data. Regardless of method, proportional interfaces have been observed to lead to higher levels of user adaptation [37], potentially due to their greater resemblance to natural motor control.

An alternative way of extending myoelectric pattern recognition into the continuous domain, that does not require continuous target measurements, is to leverage the fact that aggregated sEMG activity can be modulated volitionally, and thusly estimate movement class and intensity of contraction separately. This approach, which has been applied both in previous laboratory studies (e.g. [38–40]) and commercially [41], use a classifier to determine what gesture is to be performed. Following classification, the detected gesture is performed with velocity directly proportional to either (I) the concurrently estimated force of contraction (with e.g. instantaneous sEMG magnitude as proxy), or to (II) some monotonously increasing function thereof. Such functions can be tuned automatically and independently for each detectable movement, thereby accounting for systematic differences in intensity between movement classes [40]. Albeit uncomplicated and demonstrably effective, these strategies can be understood as problematic for a number of reasons. Firstly, there is no guarantee that the pattern associations learned during model calibration will be generalizable to all intensities of contraction [10], and thus some sEMG patterns might inadvertently be classified as patterns cooccurring with other movement classes. Such mistakes can plausibly lead to an MCI output perceived as erratic by the user. Secondly, proportionality mediated in this way is not *simultaneous* over all available DoFs, as only a single dimension of proportional information (i.e. the globally estimated intensity of myoelectric activity) is available.

In addition to developments in pattern recognition, studies of methods which are not directly based on regression or classification have demonstrated the potential of several alternative paths towards natural, simultaneous, and proportional myoelectric control. Multisite intramuscular EMG (iEMG), which can measure motor unit action potentials directly [42], has been investigated as a mechanism for direct control, and has furthermore been shown to possess functional advantages when compared to proportional pattern recognition [43]. Weakly supervised autoencoding has shown promising results in unlabelled separation of underlying sEMG signal components which can be mapped to kinematics directly [44]. Nonnegative matrix factorization has been used [45] to extract multiple simultaneous DoFs separately from rectified and filtered sEMG while retaining their respective proportionalities. Techniques for deconvoluting high-density sEMG based on models informed by neuromuscular physiology have successfully been applied towards the same end [46]. Although at the cutting edge of electrophysiology, such approaches have, possibly due to advanced modes of signal acquisition, so far mostly been constrained to the laboratory environment.

In order to aid in the pursuit of practical MCIs and to alleviate the limitations of available methods, this paper introduces a new set of methods aimed at achieving intuitive, proportional, and simultaneous myoelectric control. Concretely, the framework is constituted by a computationally lightweight neural network topology with a compatible optimization procedure, all described in detail in the Methods section. In contrast to previous frameworks based on pattern recognition, the proposed combination of techniques operates to learn nonlinear mappings from forearm sEMG to continuous and multivariate encodings of hand- and wrist kinematics, despite only being calibrated with sEMG signals labelled with categorical movement instruction stimuli. This affords the framework the advantage of regression-based approaches (i.e. proportionality) but requiring neither kinematic ground truth data nor complicated recording protocols. Additionally, by incorporating a multi-task learning formulation of the kinematic inference problem, the framework implicitly allows for independent and simultaneous control of all considered DoFs. Due to its reliance on signal representations [47] arising from supervised learning with regularizing constraints, the novel framework is referred to as myoelectric representation learning (MRL). To demonstrate the viability of MRL and to quantify differences in performance compared to the current commercial standard for pattern recognition, this paper includes experiments in which test subjects were tested for efficacy of control when using (I) MRL, and (II) pattern recognition as represented by linear discriminant analysis (LDA) [40], to perform a virtual Fitts’s law [48] type test. Furthermore, to quantify temporal deterioration of myocontrol quality, the performances of both methods were reassessed after 7 days of intermission. Interestingly, distributed representations learned by the MRL model seem insensitive to small drifts in the data-generating distribution over time, leading to a stable interface across the two usage sessions.

## Methods

### Myoelectric representation learning

In accordance with existing pattern recognition frameworks for myoelectric control via supervised learning, the proposed MRL system operates in two modes: calibration and inference. During calibration, an adaptive model is trained to approximate a mapping from sEMG to concurrent movement intent. During inference, the calibrated model is used to regress kinematics from previously unseen sEMG samples in real-time. Just as is the case with many earlier myoelectric decoding systems, the MRL system comprises a preprocessing step followed by a multi-layered feedforward artificial neural network (ANN) model. To adapt the model to the task at hand, the system requires user-specific calibration data in the form of:

1. ${{\varvec{X}}}^{cal}=\left[{{\varvec{x}}}_{1}^{cal},\cdots ,{{\varvec{x}}}_{t}^{cal},\boldsymbol{ }\cdots ,\boldsymbol{ }{{\varvec{x}}}_{T}^{cal}\right]$, where ${{\varvec{x}}}_{t}^{cal}\in {F}_{b}^{I}$ are raw sEMG voltages at time t, ${F}_{b}$ is the set of all floating-point numbers representable by $b$ bits, $I$ is the number of sEMG channels, and $T$ is the number of calibration samples.
2. ${{\varvec{Y}}}^{cal}=\left[{{\varvec{y}}}_{1}^{cal}, \cdots , {{\varvec{y}}}_{t}^{cal}, \cdots , {{\varvec{y}}}_{T}^{cal}\right]$, where ${{\varvec{y}}}_{{\varvec{t}}}^{cal}\in {\{-\mathrm{1,0},1\}}^{J}$ is a ternary DoF-wise categorical encoding of the movement intent of the subject at time $t$, and $J$ is the number of DoFs which the system is intended to control.

Following calibration, the system can infer DoF-wise continuous output $\widehat{{\varvec{y}}}\in {F}_{b}^{J}$, corresponding to concurrent kinematics, from any time slice ${\varvec{x}}\in {F}_{b}^{I}$ of a provided sEMG time series. During inference time, the system provides an output control signal with update rate identical to that with which the input sEMG is provided.

Two principal modifications distinguish the properties of the ANN models employed here from those of previous applications of Deep Learning for the purpose of decoding myoelectric signals. The first modification, to which the network topology itself is subject, can be viewed through the prism of hard parameter sharing as known from the literature on multitask learning [49]*.* This modification, which is elaborated upon in the section titled ‘Neural Network Topology’, is the basis for the simultaneity of control enabled by calibrated models. The second modification, which involves the optimization procedure and the appertaining loss function which selects the free parameters of any instantiation of the topology, can be viewed through the prism of contractive regularization, as known from the study of deep autoencoders [50]*.* This technique, which has been reformulated for the intended purpose and is presented in the section titled ‘Calibration’, allows for proportionality of control when deploying calibrated models.

#### Preprocessing

Prior to instantiation and optimization of the neural model, the raw I-channel sEMG signals ${{\varvec{X}}}^{cal}\in {F}_{b}^{I\times T}$ of the calibration data set is subject to envelope extraction, rescaling, clipping, and nonlinear transformation: Initially, channel-wise signal envelopes are extracted from ${{\varvec{X}}}^{cal}$ by full-wave rectification and digital LTI lowpass filtering with a gain-free causal FIR filter (moving average) with length W samples. The resulting (unbounded and nonnegative) envelopes ${{\varvec{E}}}^{u}\in {F}_{b}^{I\times T}$ are thereafter linearly rescaled elementwise via (1).

1 $${E}_{i,l}^{r} \leftarrow \frac{{E}_{i,l}^{u}-{p}_{i}^{1\%}}{{p}_{i}^{99\%}-{p}_{i}^{1\%}}$$

Here, ${p}_{i}^{1\mathrm{\%}}$ and ${p}_{i}^{99\mathrm{\%}}$ are the 1st and 99th percentile, respectively, of the recorded voltages of the $i$ th sEMG channel envelope across all $T$ samples of ${{\varvec{E}}}^{u}$. The resulting rescaled signals ${{\varvec{E}}}^{r}\in {F}_{b}^{I\times T}$ are subject to clipping and transformed by the element-wise square root operator as shown in (2).

2 $${E}_{i,l}^{cal} \leftarrow \sqrt{max(0,min(1,{E}_{i,l}^{r}))}$$

These steps: (I) ensure that all elements of the obtained matrix ${{\varvec{E}}}^{cal}=\left[{{\varvec{e}}}_{1}^{cal}\boldsymbol{ }\cdots \boldsymbol{ }{{\varvec{e}}}_{t}^{cal}\boldsymbol{ }\cdots \boldsymbol{ }{{\varvec{e}}}_{T}^{cal}\right]\in {F}_{b}^{I\times T}$ (which are to be used as ANN inputs) are constrained to the interval [0,1]; (II) mitigate the influence of outlier samples in ${{\varvec{X}}}^{cal}$; and (III) provide implicit threshold values under which the envelopes are taken to equal zero. The square root operator is included to bias resolution towards high levels of muscle contraction by smoothing variations in signal envelopes at values close to the observed maximum. Due to the lack of time shifts introduced in the process of generating ${{\varvec{E}}}^{cal}$ from ${{\varvec{X}}}^{cal}$, the columns (i.e. time points) of the ground truth movement intent matrix ${{\varvec{Y}}}^{cal}$ are synchronous with those of ${{\varvec{E}}}^{cal}$.

When the MRL system operates in inference mode, acquired sEMG samples are processed in an identical manner by utilizing online filtering and rescaling with the calibration data statistics ${{\varvec{p}}}^{1\%}=\left[{p}_{1}^{1\%},\boldsymbol{ }\cdots ,\boldsymbol{ }{p}_{i}^{1\%},\boldsymbol{ }\cdots ,\boldsymbol{ }{p}_{I}^{1\%}\right]$ and ${{\varvec{p}}}^{99\%}=\left[{p}_{1}^{99\%},\boldsymbol{ }\cdots ,\boldsymbol{ }{p}_{i}^{99\%},\boldsymbol{ }\cdots ,\boldsymbol{ }{p}_{I}^{99\%}\right]$ as per Eqs. (1) and (2).

#### Neural network topology

**Fig. 1:**
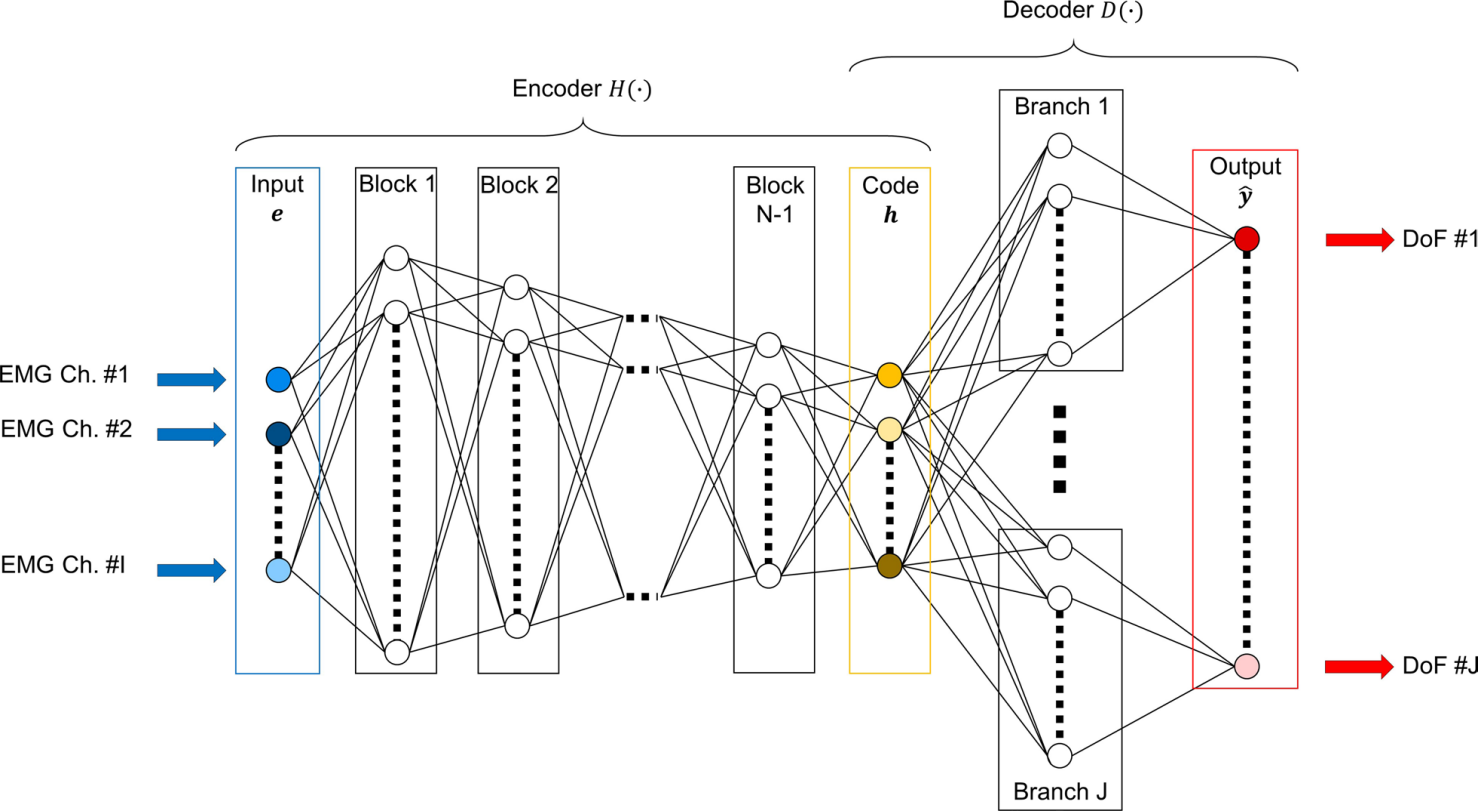
Schematic overview of the regression feedforward neural network topology central to the MRL framework. A time slice ${\varvec{e}}$ of the signal envelopes of $I$-channel sEMG at time $t$ is fed through an *encoder* network, constituted by $N$ fully connected blocks, which transforms ${\varvec{e}}$ into an alternative *representation*, i.e. code, ${\varvec{h}}$. A set of $J$ *decoder* networks, each constituted by a single hidden block, decode this representation to independently estimate the activations of $J$ DoFs, interpreted collectively as the proportional and simultaneous output command $\widehat{{\varvec{y}}}$ corresponding to the movement intent of the user at time $t$

A network topology of the kind described in this section is depicted in Fig. 1. The ANN model takes as input an sEMG envelope time slice ${\varvec{e}}={\left[{e}_{1}\boldsymbol{ }\cdots \boldsymbol{ }{e}_{i}\boldsymbol{ }\cdots \boldsymbol{ }{e}_{I}\right]}^{{\varvec{T}}}$, obtained as described above, and provides a numeric representation of inferred concurrent kinematics $f({\varvec{e}})=\widehat{{\varvec{y}}}\in {\left[{\widehat{y}}_{1}\boldsymbol{ }\cdots \boldsymbol{ }{\widehat{y}}_{j}\boldsymbol{ }\cdots \boldsymbol{ }{\widehat{y}}_{J}\right]}^{{\varvec{T}}}$ as output. Although the ANN models of the current study are instantiated and calibrated end-to-end (as will be described in the next section), the mappings they represent can naturally be understood as processes constituted by two elements in succession: encoding followed by decoding. Initially, **e** is mapped to the input layer which in turn is fed through $N$ blocks, the sequence of which constitute the encoder network said to be performing the function $H(\cdot )$. (The depth $N$ of the decoder network is a hyperparameter to be selected prior to network instantiation.) Internally, each such encoder block is constituted by a fully connected layer [18] followed by a leaky rectifier linear unit (ReLU) activation layer [51], whose output in turn is subject to layer normalization [52] without learnable parameters. The numbers of output neurons of the first fully connected block of the encoder network is here set to equal ${2}^{K}$, where $K\ge N$ is a model hyperparameter. The number of output neurons for each consecutive encoder block is set to equal half of that of its predecessor, and the number of output neurons at the last encoder block thus equals ${2}^{K-N+1}$. This output of this last encoder block, referred to as the code $H({\varvec{e}})={\varvec{h}}\in {F}_{b}^{K-N+1}$, is subsequently fed into a set of parallel decoder sub-networks, collectively said to be performing the function $D(\cdot )$. These decoder sub-network, each associated with one of the $J$ decodable DoFs, are constituted by a single fully connected hidden block (again incorporating leaky ReLU activations and parameter-free layer normalization) of output size ${2}^{S}$ ($S$ being a hyperparameter) and a terminal fully connected layer with one output neuron with linear activation function. The resulting concatenation of values $\{{\widehat{y}}_{1},\dots ,{\widehat{y}}_{J}\}$, each of which is the output of one of the decoder networks, constitute the network output, i.e. $f({\varvec{e}})=D(H({\varvec{e}}))=D({\varvec{h}})=\widehat{{\varvec{y}}}={\left[{\widehat{y}}_{1}, \cdots , {\widehat{y}}_{j}, \cdots , {\widehat{y}}_{J}\right]}^{T}$, where ${\widehat{y}}_{j}$ is to be interpreted as the inferred ‘intensity’ of movement intent for the $j$ th kinematic output DoF.

In previous work aimed at achieving simultaneous (i.e. multi-DoF) myoelectric control via the use of regression ANNs, a distinction is sometimes made between the use of shared models and dedicated models [36, 45]. Shared models here refer to a type of network where each DoF to be inferred is represented as a single neuron in the output layer. Dedicated models, in contrast, use one separate network per DoF, each with a single output neuron. Within this framework, it has typically been found that dedicated models outperform shared models in tasks of multivariate kinematic regression [53]. The novel ANN topology used in the current study can be construed as a hybrid between these two extremes: The encoder $H(\cdot )$ is shared between the DoFs and the decoder $D(\cdot )$ is formed by dedicated branch networks.

Use of an initial shared network followed by multiple task-specific subnetworks, sometimes referred to as hard parameter sharing [49], has previously been studied in the context of multitask learning, where the simultaneous estimation of multiple related target variables often results in better performance for all estimations individually [54]. It is thus hypothesised that the alternative presented here is an improvement over the dedicated model approach: The encoder can learn to transform sEMG into a more useful signal representation ${\varvec{h}}$ for the purpose of decoding all DoFs, while at the same time allowing for the advantages of using dedicated subnetworks to infer the activation strengths of all DoFs separately.

#### Calibration

The calibration of the MRL neural network topology previously presented, i.e. the tuning of model parameters (weights ${\varvec{W}}$ and biases ${\varvec{b}}$) for a given user of the system, is specified by two components: (I) a differentiable loss metric $\mathcal{L}=\mathcal{L}({{\varvec{E}}}^{cal},{{\varvec{Y}}}^{cal},{\varvec{\theta}})$, where ${\varvec{\theta}}=\{{\varvec{W}},{\varvec{b}}\}$ is the complete set of free model parameters, and (II) an iterative, gradient descent procedure for its minimization with respect to the parameter values constituting ${\varvec{\theta}}$.

The loss metric $\mathcal{L}$, which is to be minimized, is given below in (3) and is defined as a weighted sum of the two loss functions ${\mathcal{L}}_{i}$ and ${\mathcal{L}}_{c}$.

3 $$\mathcal{L}={\mathcal{L}}_{i}+{\mathrm{\alpha }}_{c}{\mathcal{L}}_{c}$$

The value of ${\alpha }_{c}\in {F}_{b}$ is a hyperparameter representing the relative weighting of the two sources of error. The first term ${\mathcal{L}}_{i}$ in the definition above is referred to as the inference loss and is given in (4).

4 $${\mathcal{L}}_{i}\left({{\varvec{E}}}^{cal},{{\varvec{Y}}}^{cal},{\varvec{\theta}}\right)=\frac{1}{T}\sum_{t}^{T}{\Vert {{\varvec{y}}}_{{\varvec{t}}}^{cal}-{\widehat{{\varvec{y}}}}_{{\varvec{t}}}\Vert }_{1}$$

Here ${\Vert \cdot \Vert }_{1}$ denotes the ${L}_{1}$ vector norm, i.e. ${\Vert {\varvec{v}}\Vert }_{1}={\sum }_{i}\left|{v}_{i}\right|$. This loss term, which is functionally identical to DoF-wise mean absolute error, quantifies the discrepancy between ground truth movement intent ${{\varvec{y}}}_{{\varvec{t}}}^{cal}$ and the corresponding value ${\widehat{{\varvec{y}}}}_{{\varvec{t}}}=G(H({{\varvec{e}}}_{t}^{cal}))$ inferred by the ANN model, averaged across all calibration example instances. Importantly, in contrast to the more commonly used mean square error of linear regression fame, ${\mathcal{L}}_{i}$ penalizes DoF-wise distance from estimate to target linearly. When deployed in conjunction with the topology presented above, ${\mathcal{L}}_{i}$ can additionally be understood as a form of regularization in and of itself: In order to achieve a low value of ${\mathcal{L}}_{i}$, the model needs to accurately infer the activation of all $J$ DoFs simultaneously. As has been observed for multitask learning models in general [54], multiple related sub-tasks of this kind impose regularizing constraints onto each other, impacting which kinds of data representations are likely to arise throughout the network and leading to a synergistic effect whereby the performance on every sub-task (i.e. the estimation quality for every individual DoF) is expected to improve.

**Fig. 2:**
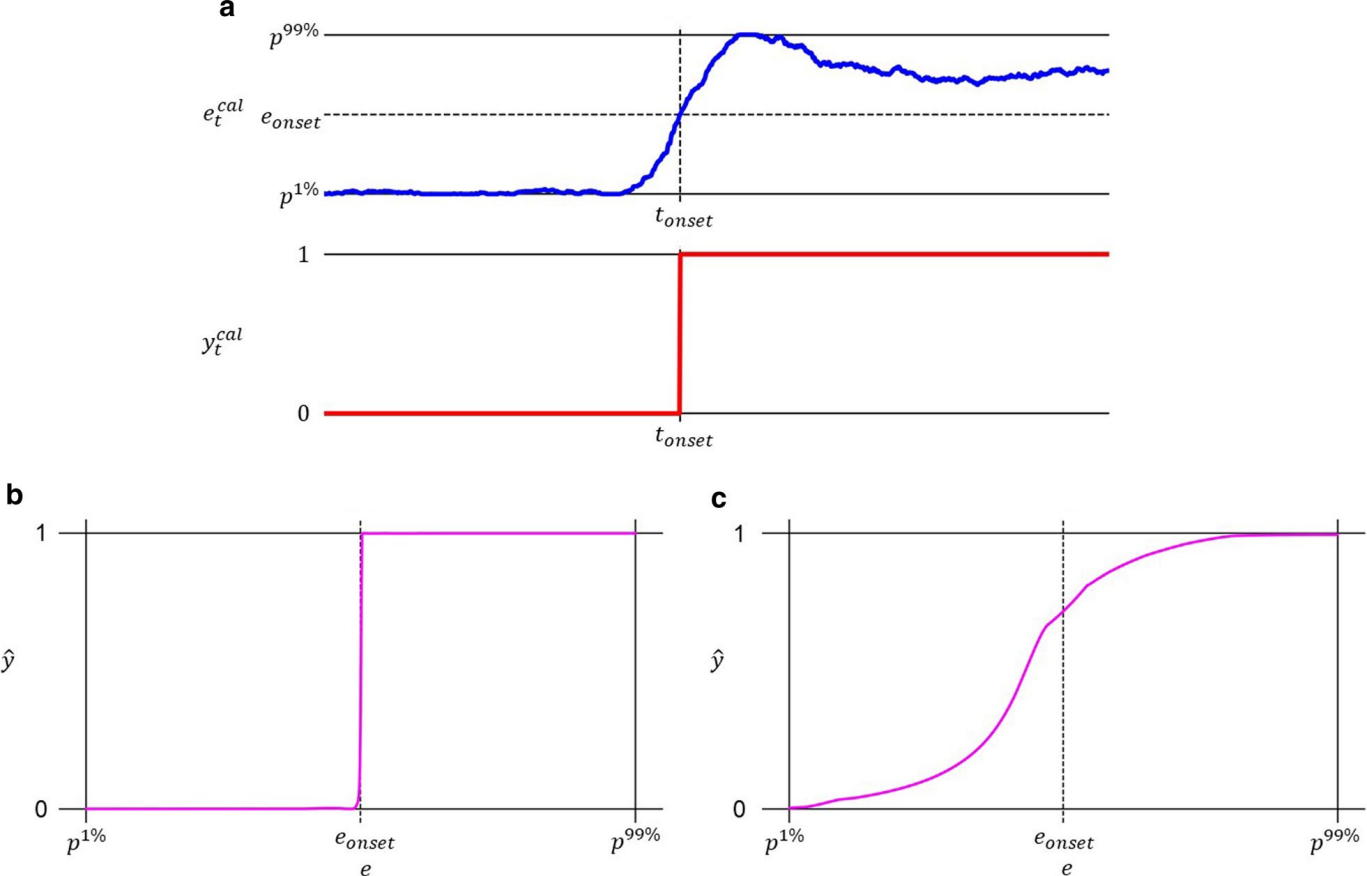
The effect of contractive regularization on proportionality of control. The effect is illustrated by a simplified example with univariate regressor ${\varvec{e}}=e$ and regressand ${\varvec{y}}=y$. **a** Plot of calibration data, simulating movement onset, constituted by the sEMG envelope ${e}_{t}^{cal}$ with $I=1$ channel (upper blue) and the concurrent movement instruction ${y}_{t}^{cal}$ with $J=1$ DoF (lower red). **b** Plot of the learned mapping $\widehat{y}={f}_{1}(e,{\varvec{\theta}})$ performed by an MRL network calibrated by minimizing ${\mathcal{L}}_{i}$ with respect to ${\varvec{\theta}}$. This model approximates a categorical decision threshold and does not enable proportionality. **c** Plot of the learned mapping $\widehat{y}={f}_{2}(e,{\varvec{\theta}})$ performed by an MRL network calibrated by instead minimizing ${\mathcal{L}}_{i}+{\mathrm{\alpha }}_{c}{\mathcal{L}}_{c}$ with respect to ${\varvec{\theta}}$. This model produces output which varies smoothly with latent muscle activity and thus enables proportionality

Albeit clearly a necessary condition for desirable model behaviour, a low value of ${\mathcal{L}}_{i}$ is not a sufficient goal of the optimization procedure in the context of kinematic estimation. To realize this fact, one can consider the case of a model which has learned to infer only categorical output (i.e. $\widehat{{\varvec{y}}}\in {\{-\mathrm{1,0},1\}}^{J}$) that perfectly matches the provided categorical targets in ${{\varvec{Y}}}^{cal}$ (e.g. the model in Fig. 2b). Furthermore, assume this model performs well only with input sEMG envelopes generated at intensities of muscle contraction identical to those employed during the collection of the calibration data ${{\varvec{E}}}^{cal}$. Such a model, although clearly deficient for the task at hand, would achieve very low values of ${\mathcal{L}}_{i}$. To generate calibrated models which in addition enable the previously stated goal of proportionality of control, as well as achieve adequate performance with previously unobserved contraction intensities, the total loss function contains a second, regularizing term, referred to as the contractive loss, denoted by ${\mathcal{L}}_{c}$, and defined in (5).

5 $${\mathcal{L}}_{c}({{\varvec{E}}}^{cal},{\varvec{\theta}})=\frac{1}{TIJ}\sum_{t}^{T}\sum_{i}^{I}\sum_{j}^{J}{\left({\left.\frac{\partial {\widehat{y}}_{j}}{\partial {e}_{i}}\right|}_{{e}_{i}={{\varvec{E}}}_{{\varvec{t}},{\varvec{i}}}^{{\varvec{c}}{\varvec{a}}{\varvec{l}}}}\right)}^{2}$$

${\mathcal{L}}_{c}$ here represents the squared gradient of every individual output element ${\widehat{y}}_{j}$ with regard to every individual input element ${e}_{i}$, computed at the value generated from the presented inputs (i.e. the columns of ${{\varvec{E}}}^{cal}$) and averaged across all such provided calibration examples. Stated equivalently, ${\mathcal{L}}_{c}$ is Frobenius norm of the Jacobian matrix of the multivariate function performed by (the current parameter configuration of) the network [18], averaged across all calibration example instances $\{{{\varvec{e}}}_{t}^{cal},{{\varvec{y}}}_{t}^{cal}\}$, $t\in [1,T]$.

To understand the consequences of a minimized value of ${\mathcal{L}}_{c}$, it is important to note that the mapping performed by any ANN model with fully differentiable activation functions (i.e. compatible with backpropagation) is, by definition, continuous in the mathematical sense [18]. However, without any constraints, such a model may still learn parameter values ${\varvec{\theta}}$ such that the gradient $\partial {\widehat{y}}_{j}/\partial {e}_{i}$ of any element ${\widehat{y}}_{j}$ in the output with regard to any element ${e}_{i}$ in the input becomes arbitrarily large at any number of points in the space of possible inputs. To counteract this sometimes unwanted property by ‘incentivizing’ models to learn to associate limited deviations in input with only limited deviation in output, contractive loss of the same formulation as that of (5) has previously been applied as a form of regularization for the class of unsupervised neural network models known as *contractive autoencoders* [50].

The postulated relevance of ${\mathcal{L}}_{c}$ in the current problem formulation lies in the common assumption that muscle kinetics, and by extension limb kinematics, correlate (nonlinearly) with the concurrent signal envelope as extracted from sEMG collected at relevant recording sites [40]. Formulated in the MRL framework, this assumption can be stated differently: increases or decreases of the values constituting $\widehat{{\varvec{y}}}$ should only occur in conjunction with, and (approximately) monotonically with, increases and decreases, respectively, of the values constituting ${\varvec{e}}$. A network with this property will both give rise to proportionality of control output and achieve a small value for ${\mathcal{L}}_{c}$, thereby warranting the inclusion of this loss term here. A specific example illustrating how minimizing ${\mathcal{L}}_{c}$ induces proportionality can be viewed in Fig. 2.

With the total loss $\mathcal{L}$ established as above, its minimization with respect to ${\varvec{\theta}}$ is performed iteratively via error backpropagation and the AdamW algorithm [55] (requiring hyperparameters $\upeta$, ${\beta }_{1}$, ${\beta }_{2}$, and weight decay $\lambda$) for gradient descent in minibatches of size $B$ (a hyperparameter). The minibatch affiliation of each individual calibration example $\{{{\varvec{e}}}_{t}^{cal},{{\varvec{y}}}_{t}^{cal}\}$ is determined randomly at the start of every training epoch. All network weights ${\varvec{W}}$ are initialized randomly via Glorot initialization [56] and all biases ${\varvec{b}}$ are initially set to 0.

Prior to being fed into the ANN model for loss evaluation and parameter updating, the input sEMG envelope time slices of each minibatch is at every iteration corrupted additively with isotropic white Gaussian noise of predefined variance ${\sigma }^{2}$ (a hyperparameter). This step, inspired by its analogue in denoising autoencoders [57], has a twofold purpose: Firstly, it acts as a form of data augmentation, whereby the model overfitting to spurious patterns in the calibration data is made more unlikely [18]. Secondly, an ANN model calibrated with such input data will learn to map elements close to each other in the space of possible inputs ${\varvec{e}}$ to elements close to each other in the space of possible outputs ${\varvec{y}}$ [57]. In conjunction with the minimization of the contractive loss ${\mathcal{L}}_{c}$, this effect contributes to calibrated models which has the here desired property of proportionality.

Before the calibration process begins, a percentage $P$ (a hyperparameter) of the available calibration data $\{{{\varvec{E}}}^{cal},{{\varvec{Y}}}^{cal}\}$ is sampled randomly, without replacement, to be held out and used as validation data ${\{{\varvec{E}}}^{val},{{\varvec{Y}}}^{val}\}$. At the conclusion of every Adam update iteration, this validation data is used to compute a validation error ${\mathcal{L}}_{v}=\mathcal{L}({{\varvec{E}}}^{val},{{\varvec{Y}}}^{val},{\varvec{\theta}})$ of the model parameter configuration ${\varvec{\theta}}$ at that iteration. The optimization process continues until ${\mathcal{L}}_{v}$ is greater than that obtained $V$ iterations previously (i.e. a form of early stopping) or until a total of $M$ parameter update iterations have been performed, whichever comes first ($M$ and $V$ are hyperparameters). At this point, the model is considered calibrated and can be used for real-time inference of continuously encoded movement intent (i.e. kinematics).

### Benchmark myoelectric pattern recognition framework

For the purpose of verifying the conjectured advantages of using MRL for myoelectric control, a proportional myocontrol pattern recognition method based on LDA is here chosen as the object of comparison due to its paradigmatic role in contemporary and commercially available prosthetic systems [41]. Implementation details and hyperparameter values are selected to be identical to those of Method 2 introduced by Scheme et al*.* in [40], as this approach, like MRL, only requires sEMG collected at a single level of muscle contraction for calibration. Furthermore, the selected method has been used in clinical settings for some time and thus represents a reliable application of pattern recognition for myoelectric control. For brevity, the method for motion-normalized proportional control denoted Method 2 in [40] will in its entirety henceforth be referred to simply as LDA. Importantly, LDA does not require any manual tuning of parameters (e.g. gains and thresholds), thus eliminating the risk that the experiment supervisor impacts the quality of the calibration results in the current study. A brief summary of this method is provided below.

In contrast to MRL, LDA does not operate on sEMG envelopes alone; instead, a sliding window is used to extract 4 time-domain features per channel from the raw sEMG signals: Mean Absolute Value (MAV), Zero Crossings, Slope Sign Changes, and Waveform Length, all introduced by Hudgins et al*.* in [12]. As LDA is not inherently simultaneous over the available DoFs, each detectable movement combination is instead assigned a unique categorical value to be inferred in order to allow for multiarticulate control. For a problem formulation involving J independently controllable, bidirectional DoFs, LDA thus operates to map each processed feature time window to a member of the set of ${3}^{J}$ possible movement classes (each bidirectional and categorical DoF can independently assume 3 mutually exclusive states). LDA achieves proportionality by computing a proportionality scalar ${\mathrm{PC}}_{m}$, contingent on the index $m\in \left[0,{3}^{J}-1\right]$ of the currently inferred movement class, to each feature window. ${\mathrm{PC}}_{m}$ is computed in parallel with the running classification process by use of the MAV feature as detailed in (6), (7), and (8).

6 $${\mathrm{PC}}_{m}={\left(\frac{1}{{C}_{m}}\sum_{i=1}^{I}{S}_{i,m}{\mathrm{MAV}}_{i}\right)}^{2}$$

7 $${C}_{m}=\sum_{i=1}^{I}{S}_{i,m}$$

8 $${S}_{i,m}=\frac{1}{{K}_{m}}\sum_{k=1}^{{K}_{m}}{\mathrm{MAV}}_{i,m,k}^{cal}$$

${\mathrm{MAV}}_{i}$ is the MAV feature value of the $i$ th sEMG channel of the processed window,${\mathrm{MAV}}_{i,m,k}^{cal}$ is the MAV feature value of the $i$ th sEMG channel of the $k$ th feature window of the $m$ th movement of the calibration data, and ${K}_{m}$ is the total number of calibration data feature windows for movement $m$. As before, $I$ is the number of sEMG channels. The training of the classification algorithm, together with the computation of the class centres ${\varvec{C}}=\left[{C}_{1}\boldsymbol{ }\cdots \boldsymbol{ }{C}_{m}\boldsymbol{ }\cdots \boldsymbol{ }{C}_{M}\right]$ and of the matrix ${\varvec{S}}=\{{S}_{i,m}\}$, constitute the entirety of the LDA calibration which is performed on a subject-wise basis.

### Experiments

#### Subjects

20 able-bodied subjects (age range 24–58 years, median age 32 years, 13 male, 7 female, 18 right-handed, 2 left-handed) without history of known neuromuscular or musculoskeletal disorders participated in the current study. The study was approved by the Regional Ethics Review Board in Lund, Sweden and was conducted in accordance with the tenets of the Declaration of Helsinki. All subjects were informed about the contents of the experiments, both verbally and in writing, and gave their informed and written consent.

Each subject participated in two separate experiment sessions: the first session, hereinafter referred to as day 1, consisted of both calibration data acquisition, model calibration, and evaluation of the two methods (MRL and LDA) for myoelectric control. This first session lasted for a total duration of 1 h or less, with some variations across subjects. The second session, carried out one week later and hereinafter referred to as day 7, entailed evaluation of both methods without any recalibration (in order to quantify interface stability) and thus lasted for a shorter duration (approximately 30 min). None of the subjects had prior experience with the studied myoelectric control methods.

#### Calibration data acquisition

**Fig. 3:**
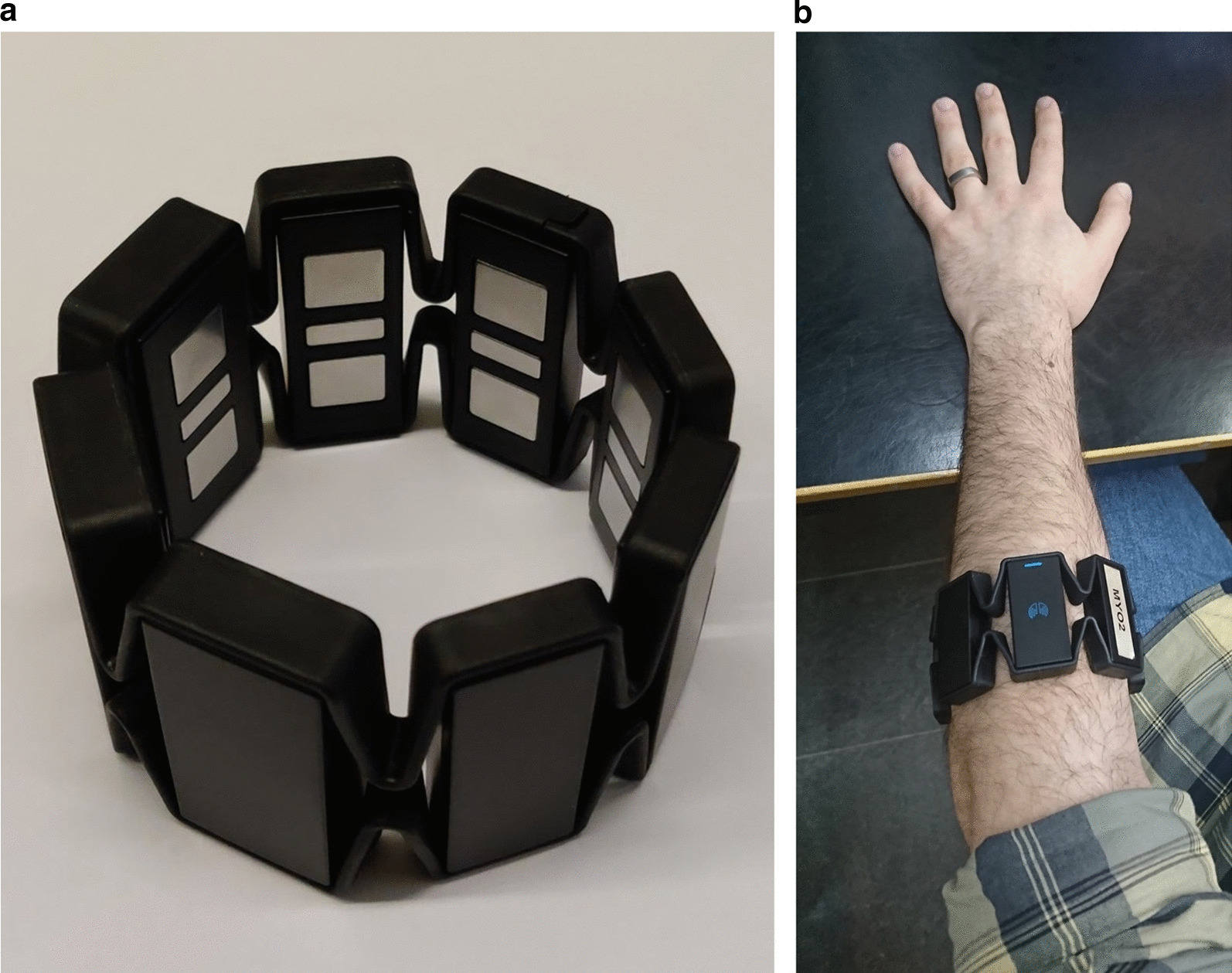
The acquisition setup. Photographs depict **a** the Myo armband used for recording sEMG signals and **b** the placement of the Myo armband on the arm of a subject

A Myo armband (Thalmic Labs, Canada), composed of 8 circularly arranged dry surface electrodes of size 100 mm^2^, was used to acquire sEMG time series from subjects (see Fig. 3a). Prior to A/D conversion, EMG voltage signals were filtered using a built-in analogue bandpass filter with passband 5–100 Hz and a 50 Hz notch filter. Digital signals were sampled with 8-bit precision at a rate of ${F}_{s}=200$ Hz and transferred at that same rate to a host desktop computer (on which all signal processing was performed) wirelessly via Bluetooth. The Myo armband was placed enclosing the dominant forearm of the subject at a level approximately 1/3 of the distance from the elbow joint to the wrist joint (see Fig. 3b). A photograph depicting the armband position and orientation was taken on day 1 in order to provide guidance for the redonning on day 7. The process of donning the armband could always be completed in a time of at most one minute. Following the placement of the armband, the subject was seated comfortably at approximately $1$ m distance from a computer screen with the elbow resting on a table placed in front of the subject. The subjects could freely vary the position and angle of the elbow joint during all parts of the experiment.

The current study concerned the independent control of 2 separate DoFs: flexion and extension of the wrist (communicated to subjects as ‘wrist right’ and ‘wrist left’, contingent on the handedness of the subject), and flexion and extension of all digits simultaneously (communicated to subjects as ‘hand close’ and ‘hand open’, respectively). A ternary movement encoding approach was employed in the current study, resulting in ${3}^{2}=9$ possible compound movements (listed in Table 1).

**Table 1:** The calibration movements for right-handed subjects, all recorded on day 1

Movement class	Description	Ternary movement encoding
$m=0$	Rest	${\varvec{y}}=[0, 0]$
$m=1$	Wrist flexion	${\varvec{y}}=[-1, 0]$
$m=2$	Wrist extension	${\varvec{y}}=[1, 0]$
$m=3$	Flexion of the digits	${\varvec{y}}=[0,-1]$
$m=4$	Extension of the digits	${\varvec{y}}=[0, 1]$
$m=5$	Wrist flexion and Flexion of the digits	${\varvec{y}}=[-1, -1]$
$m=6$	Wrist flexion and extension of the digits	${\varvec{y}}=[-1, 1]$
$m=7$	Wrist extension and flexion of the digits	${\varvec{y}}=[1, -1]$
$m=8$	Wrist extension and extension of the digits	${\varvec{y}}=[\mathrm{1,1}]$

Prior to calibration data acquisitions, sEMG was recorded while the subject performed all movements, excluding rest, with maximum voluntary contraction for 5 s. This step served to familiarize the subject with the movement combinations under consideration and was used to compute a maximum voluntary contraction (MVC) signal magnitude value specific to each movement by summing the MAV over all 8 sEMG channels.

**Fig. 4:**
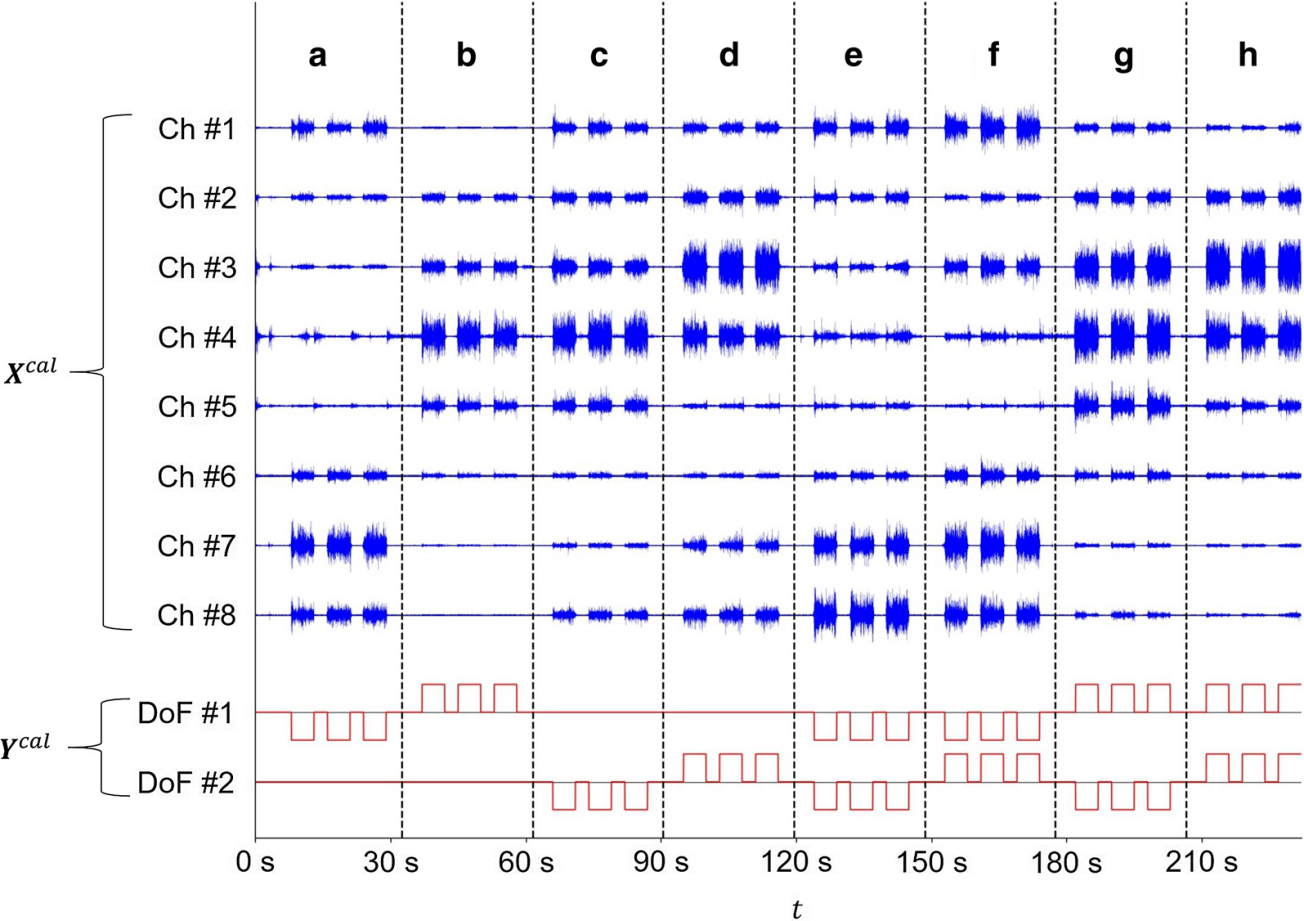
Calibration signals acquired from a representative, right-handed subject. The calibration data is constituted by raw sEMG (${{\varvec{X}}}^{cal}$) and trinary ($-1$, $0$, or $1$) DoF- and sample-wise encodings of concurrent movement instruction stimuli (${{\varvec{Y}}}^{cal}$). **a** Wrist flexion. **b** Wrist extension. **c** Flexion of the digits. **d** Extension of the digits **e** Wrist flexion and flexion of the digits. **f** Wrist flexion and extension of the digits. **g** Wrist extension and flexion of the digits. **h** Wrist extension and extension of the digits

Calibration data was recorded via an automated acquisition program which prompted the subject to perform all nonrest movements for 3 repetitions, each lasting for a duration of 5 s and separated by 3 s of rest. To aid the subject in applying a sustainable and consistent level of contraction across movements, the MAV of the EMG signal, extracted via a sliding window of length 0.5 s and summed over all channels, was mapped to the height of a bar shown in real-time on the computer screen together with a threshold set to equal 50% of the movement-specific MVC magnitude computed earlier; subjects were instructed to keep the height of the bar as close to the threshold as possible. Once the program was concluded, recorded sEMG was saved together with synchronized movement instruction stimuli signals. An example of such calibration data from a single subject is shown in Fig. 4.

#### Calibration of models

The MRL model and the LDA model described previously were both automatically calibrated immediately following the conclusion of the data acquisition phase. The methods were executed with Python 3.6, using the SKLearn library [58] to implement the LDA model and the TensorFlow [59] library to implement the MRL model. No part of the calibration of either method required any manual intervention by the experiment supervisor.

The hyperparameter of the MRL framework had been selected empirically based on data from pilot work with subjects who were not participating in the current study and were not subject to change for the duration of the experiments; exact numerical values are presented in Table 2. Analysis of the ANN topology resulting from this configuration using the computational cost estimation heuristics from [27] revealed an approximate inference-mode time complexity of 5.5 MFLOPS (at 200 forward passes per second, i.e. synchronously with sEMG sampling) and a memory footprint of 59.5 kB (excluding the computational cost of preprocessing); these are requirements compatible with contemporary embedded systems. Furthermore, the use of a preprocessing filter length of $W=0.5$ s (100 samples at ${F}_{s}=200$ Hz) corresponds to a group delay (i.e. lag) of $(100-1)/(2{F}_{s})=0.2475$ s for a FIR filter [60]—less than the typically desired maximum delay of 300 ms in myocontrol applications [61].

**Table 2:** The hyperparameters of the MRL framework and their respective values selected for the current study

Hyperparameter	Symbol	Value
Floating point precision	$b$	$32$ bits
Number of sEMG channels	$I$	$8$
Number of decodable DoFs	$J$	$2$
Envelope extraction filter length	$W$	$0.5$ s
Size of first encoder layer	${2}^{K}$	$128$
Encoder network depth	$N$	$5$
Code size	${2}^{K-N+1}$	$8$
Decoders hidden layer size	${2}^{S}$	$32$
Contractive loss weigh	${\alpha }_{c}$	${10}^{-2}$
Adam hyperparameters	$\upeta$	${10}^{-4}$
	${\beta }_{1}$	$0.9$
	${\beta }_{2}$	$0.999$
Corruptive noise variance	${\sigma }^{2}$	${10}^{-1}$
Weight decay	$\lambda$	${10}^{-6}$
Minibatch size	$B$	${2}^{12}$
Validation set percentage	$P$	$10\%$
Validation lookback	$V$	$300$
Maximum number of iterations	$M$	$5000$

**Fig. 5:**
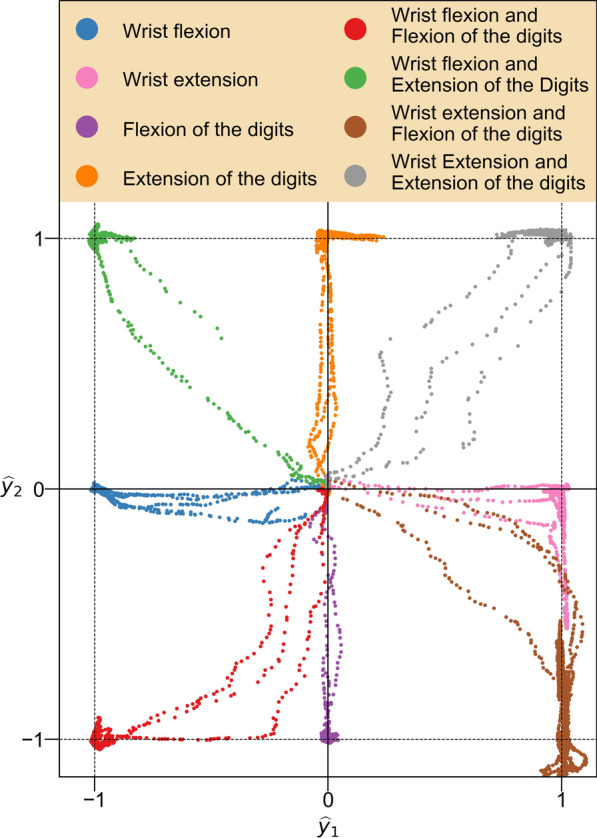
Example of MRL calibration results from a single subject. Each point represents the output $\widehat{{\varvec{y}}}={\left[{\widehat{y}}_{1}, {\widehat{y}}_{2}\right]}^{T}$ of the calibrated MRL model when fed an 8-channel sEMG sample from the calibration data, coloured according to the movement instruction stimuli with which it cooccurred. Each ‘trail’ connecting the origin to a cluster represents the onset and conclusion of a movement repetition

Calibration of the MRL model was performed according to the procedures outlined previously in the section titled ‘Calibration’. The average (across subjects) wall time required to calibrate a single MRL model with data collected from a single subject on the desktop computer (equipped with a Nvidia Titan V GPU) was measured as 97.96 s (SD 0.43 s). An illustration of the mapping performed by a calibrated MRL model is presented in Fig. 5.

LDA was calibrated as per the specifications of the previously summarized original study [40], with hyperparameter values as those of the same study, using a feature window of duration 160 ms (32 samples) and inter-window time increments of 15 ms (3 samples). The feature window was moved across the entirety of each collected EMG without regard to when individual movement repetitions started and ended. All $8\cdot 4=32$ features were individually renormalized to have zero mean and unit variance across all calibration data feature window locations. During real-time inference, features were similarly rescaled with the calibration set means and variances prior to being processed by the classifier. The ground truth movement class $m$ of each calibration data feature window was determined via a majority vote over its constituent time samples, thereby resolving ambiguities regarding the class affiliation of feature windows containing a shift in contraction level. The average (across subjects) wall time required to calibrate the LDA model to a single subject was measured as 1.31 s (SD 0.09 s).

Although previous studies (e.g. [33]) have made use of the same combination of feature set and acquisitions setup as the LDA benchmark framework of the current study, concerns can be raised over the appropriateness of the selected time-domain features due to the relatively limited sampling rate of the system (200 Hz). This, in turn, could potentially make comparisons between MRL and LDA unbalanced, as the former utilizes high frequency information to a lesser degree than the latter. To verify the absence of any such adverse effects on the LDA classifier originating from insufficient signal bandwidth, 10% of calibration examples (selected randomly) were for each subject withheld during calibration and used to compute a validation accuracy. The average (across subjects) validation accuracy obtained in this way was 92.71% (SD 4.05%). This level of performance is in line with what is expected in light of previous studies with comparable number of detectable movement classes [10], indicating that the acquisition setup provided sufficient information for the selected feature set and classifier.

#### Evaluation

Common offline measures of inferential performance (e.g. classification accuracy) have often been found to exhibit only limited correlation with the quality of myoelectric control as estimated by functional tests [10]. Efficacy of control was for this reason here instead evaluated using a type of real-time test originally introduced by Williams and Kirsch in [62] and based on Fitts’s law [48]. The test makes use of a human test subject to leverage the myoelectric control method under investigation to steer a cursor towards a set of circular targets presented to the subject on a computer screen. The subject and the control method are thereafter evaluated in unison via the calculation of a set of performance metrics; each metric aggregated over multiple subjects is assumed to constitute an informative measure of control method quality. This particular approach to the evaluation of man–machine control interfaces has been validated for use with control based on forearm myoelectricity multiple times (e.g. [38, 44]).

In the current study, the performance of each subjects with both myoelectric control methods were evaluated in sequence. To eliminate confounding effects on measured performance originating from user adaptation, 10 subjects were randomly selected to be evaluated using MRL first and then using LDA, whereas the remaining 10 subjects were selected to be evaluated using LDA first and then using MRL. All subjects were blind to the order in which the methods were presented to them. On day 1, the evaluation phase was undertaken directly following data acquisition and model calibration, with the Myo armband unmoved. On day 7, the evaluation phase followed directly after the initial redonning of the Myo armband. Evaluative tests were otherwise conducted identically on day 1 and on day 7.

#### The control interface

**Fig. 6:**
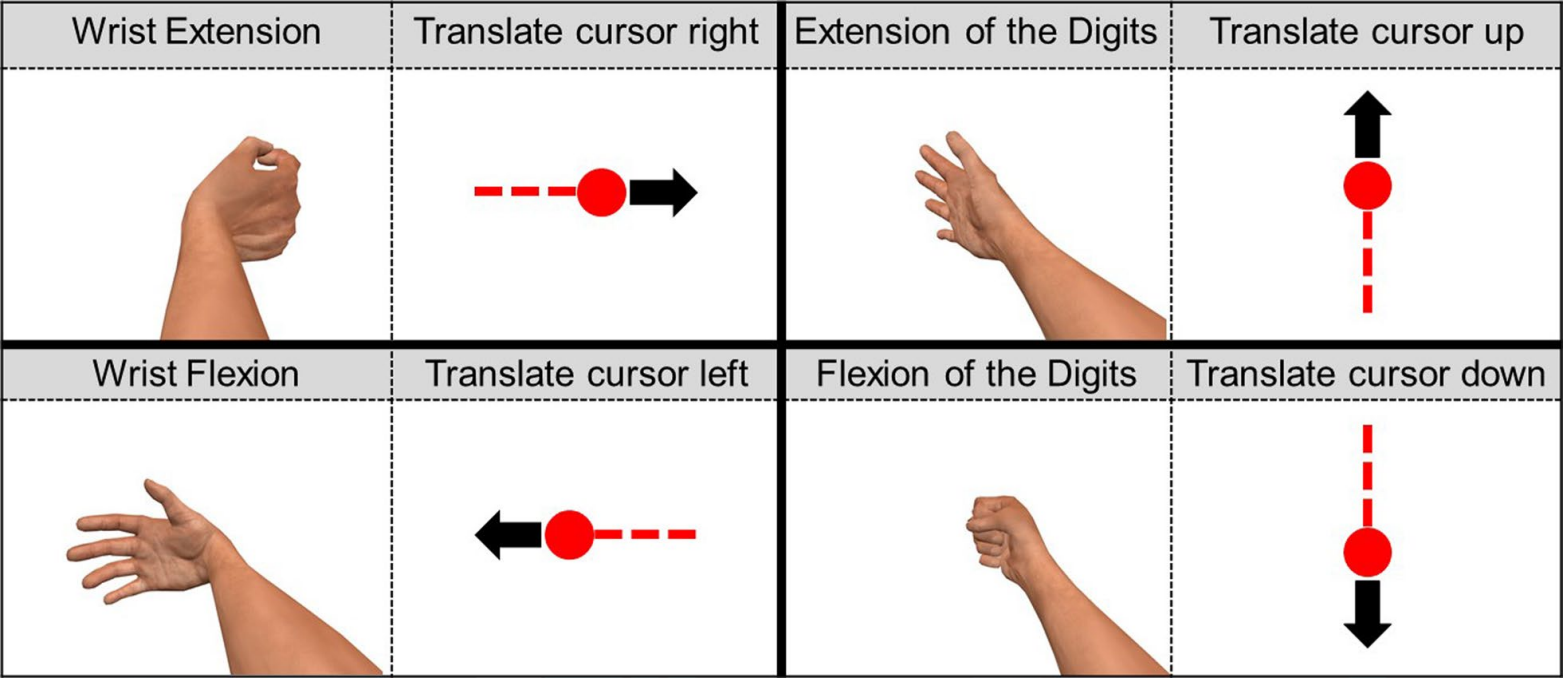
Decodable DoFs and corresponding cursor translations in the test environment for a right-handed subject. Gestures incorporating combinations of the DoFs allowed for cursor translations in intermediate directions

For both LDA and MRL, detection of wrist flexion and wrist extension corresponded to cursor translations left and right, respectively, whereas detection of flexion of the digits and extension of the digits corresponded to cursor translations down and up, respectively (as shown in Fig. 6). For LDA, the direction of cursor translation was determined according to the detected movement class, with 1-DoF movements leading to cursor translation parallel to the screen coordinate axes and 2-DoF movements leading to cursor translations parallel to the axes' diagonals. Cursor speed was scaled linearly such that ${PC}_{m}=0$ (as defined in (6)) resulted in a speed of 0 pixels per second and ${PC}_{m}=1$ resulted in a speed of 540 pixels per second. Detection of the rest class resulted in a cursor speed of 0 pixels per second. For MRL, the velocities of the cursor in the $x$- and $y$ directions were separately set to equal the first and second element of the ANN-estimated kinematics $\widehat{{\varvec{y}}}$, respectively. The cursor speed was scaled linearly such that ${\Vert \widehat{{\varvec{y}}}\Vert }_{2}=0$ resulted in a speed of 0 pixels per second and ${\Vert \widehat{{\varvec{y}}}\Vert }_{2}=1$ resulted in a speed of 540 pixels per second. Although MRL in principle allows for position control, whereby $\widehat{{\varvec{y}}}$ are mapped directly to the $x$- and $y$-coordinates of the cursor, the decision was made to consistently use this type of velocity control in order to be able to fairly compare results with those obtained with LDA.

#### Acclimation

Prior to evaluation, subjects underwent a brief acclimation exercise to become familiarized with the control interface. This preparatory step was intended to mitigate the impact of user adaptation during subsequent tests. The exercise consisted of steering the cursor towards 8 targets presented to the subject one at a time; 4 targets were placed along the $x$- and $y$ axes with peripheries touching the screen edge, and 4 targets were placed similarly at the ends of the coordinate axes' diagonals. Once each target had been reached 5 times the exercise was considered complete. The acclimation exercise, which lasted for 3–5 min, was performed prior to the Fitts’s law test on day 1 and day 7 for both MRL and LDA.

#### The performance test

**Fig. 7:**
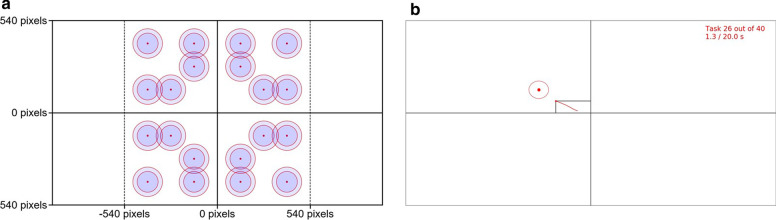
The Fitts’s law test. **a** The 40 combinations of target positions and radii selected for the current study. No target was placed on any of the coordinate axes, thus requiring the subject to activate both DoFs, either sequentially or simultaneously, for successful task completion. **b** Screen capture of the view of a subject while steering the cursor towards one of the targets presented during the test. The red ‘tail’ tracking the cursor represents the positions occupied during the preceding 0.3 s (60 samples at the 200 Hz sampling rate). A target was considered reached after a dwell time of 0.3 s, i.e. when the entirety of the tail was situated within the target periphery

Every combination of $20$ possible center coordinates (5 per coordinate system quadrant) and $2$ possible radii were selected as targets for the Fitts’s law test, resulting in $20\cdot 2=40$ unique targets (all shown in Fig. 7). The possible radii were selected as $60$ pixels and $\lceil\sqrt{2}\cdot 60\rceil=85$ pixels on a monitor with resolution $1920\times 1080$ pixels, corresponding to circular targets covering 0.6% and 1.2% of the total screen area visible to the subject, respectively.

In an order established randomly for each subject, targets were one by one plotted on the screen; the cursor was at this time held stationary at the origin. After 3 s (selected to minimize the impact of reaction time and planning), a vibration cue was given to the user through the Myo armband, and the subject was granted control of the cursor. The instruction of the subject was at this time to move the cursor to the presented target as rapidly as possible. As in previous studies [38, 44], the target was considered successfully reached once the cursor had resided within its boundary for a dwell time of 0.3 s. If 20 s passed without the subject successfully reaching the target, the task was reported as failed. Following success or failure, the target was removed from the screen, the cursor recentred, and the subject prompted to rest for 5 s, after which a new target was presented. The procedure was repeated until all 40 targets-reaching tasks had been attempted exactly once by the subject.

When all targets had either been successfully reached or failed, a set of 5 performance metrics, named completion rate ($\mathrm{CR}$), completion time ($\mathrm{CT}$), path efficiency ($\mathrm{PE}$), overshoot ($\mathrm{O}$), and throughput ($\mathrm{T}$), was calculated from the cursor trajectories traversed during the test. These metrics, defined in [62] and summarized in Table 3, characterize separate aspects of the performance of the test subject and control method across all targets. To calculate $T$, the target-specific index of difficulty ($\mathrm{ID}$) was defined as in [62] for each of the 40 targets.

**Table 3:** The real-time myocontrol performance metrics calculated for each subject and control method

Metric	Abbreviation	Summary
Completion rate [%]	$CR$	The proportion of targets which are successfully reached
Completion time [s]	$CT$	The total time from the start of the task to the target being reached, averaged across all targets (excluding failed attempts)
Path Efficiency [%]	$PE$	The ratio between the length of the optimal (straight line) path from the origin to the target and the distance traversed by the cursor, averaged across all targets
Overshoot	$O$	Total number of occurrences during the test wherein the cursor leaves the target prior to dwell time elapsion, divided by the total number of targets
Throughput [bits/s]	$T$	The ratio between the (target-wise) index of difficulty and the (target-wise) completion time averaged across all successfully reached targets

9 $$\mathrm{ID}={log}_{2}\left(\frac{D}{W}+1\right)$$

D is the Euclidean (straight-line) distance from the origin to the target and W is the diameter of the target. Each of the 40 targets were thus assigned an $ID$ out of $3\cdot 2=6$ possible values (from the 3 values of $D$ and the 2 values of $W$ visible in Fig. 7) ranging from 1.17 bits to 2.38 bits.

#### The gold standard

Once both myoelectric control methods had been evaluated on day 1, each subject was instructed to complete the aforementioned performance test using a regular computer mouse. As the computer mouse did not allow for vibration stimuli, task onset was instead accompanied by a visual trigger presented on the screen. Just as for myocontrol evaluation, targets were considered reached after a dwell time of 0.3 s (i.e. no clicking was required) and considered failed after 20 s. This step was undertaken once per test subject and was not repeated on day 7. The same set of 5 performance metrics was calculated from the obtained trajectories with the purpose of contrasting myocontrol efficacy with that of the arguably best available tool of contemporary man–machine interfacing – the performance achieved with a computer mouse during this step is for this reason denoted the gold standard (GS).

#### Statistics

The analysis described in this section was performed using SPSS Statistics version 27.0. Linear regression models with $ID$ as independent variable and target-wise completion time as dependent variable was fitted for MRL and LDA independently using all targets from all session and subjects. As in previous studies [38, 44], a high value of ${R}^{2}$ was seen as indicative of the validity of the Fitts’s law test.

Full factorial two-way repeated measures multivariate analysis of variance (MANOVA), with independent variables method (with two levels: LDA and MRL) and session (with two levels: day 1 and day 7) and all 5 performance metrics as dependent variables, was used to simultaneously assess the impact of control approach and time since calibration on aggregated control efficacy. $p$-values of less than 0.05 after Bonferroni correction for 3 estimated terms (method, session, and method*session) were considered significant. Due to the statistical significance of results obtained via MANOVA (see the Results section), this step was followed by post-hoc metric-wise univariate analysis of variance (ANOVA) in order to assess the impact of the independent variables on each metric separately. As with MANOVA, two-way full factorial repeated measure designs with a $p<0.05$ significance level (subject to Bonferroni correction for multiple comparisons [63]) were employed for this step. In addition to the $p$-values obtained via MANOVA and ANOVA, the Cohen’s $d$ effect size [64] separating MRL from LDA was computed for each metric using the full concatenation of results obtained on day 1 and on day 7.

## Results

For MRL, a strong linear relationship (coefficient of determination ${R}^{2}=0.97$ with $p=0.0010$) was observed between ID and CT. The corresponding value for LDA was computed as ${R}^{2}=0.89$ with $p=0.0019$, lending credence to a view of a Fitts’s law test as suitable for method evaluation and by extension the appropriateness of throughput $T$ as a measure of overall performance.

**Fig. 8:**
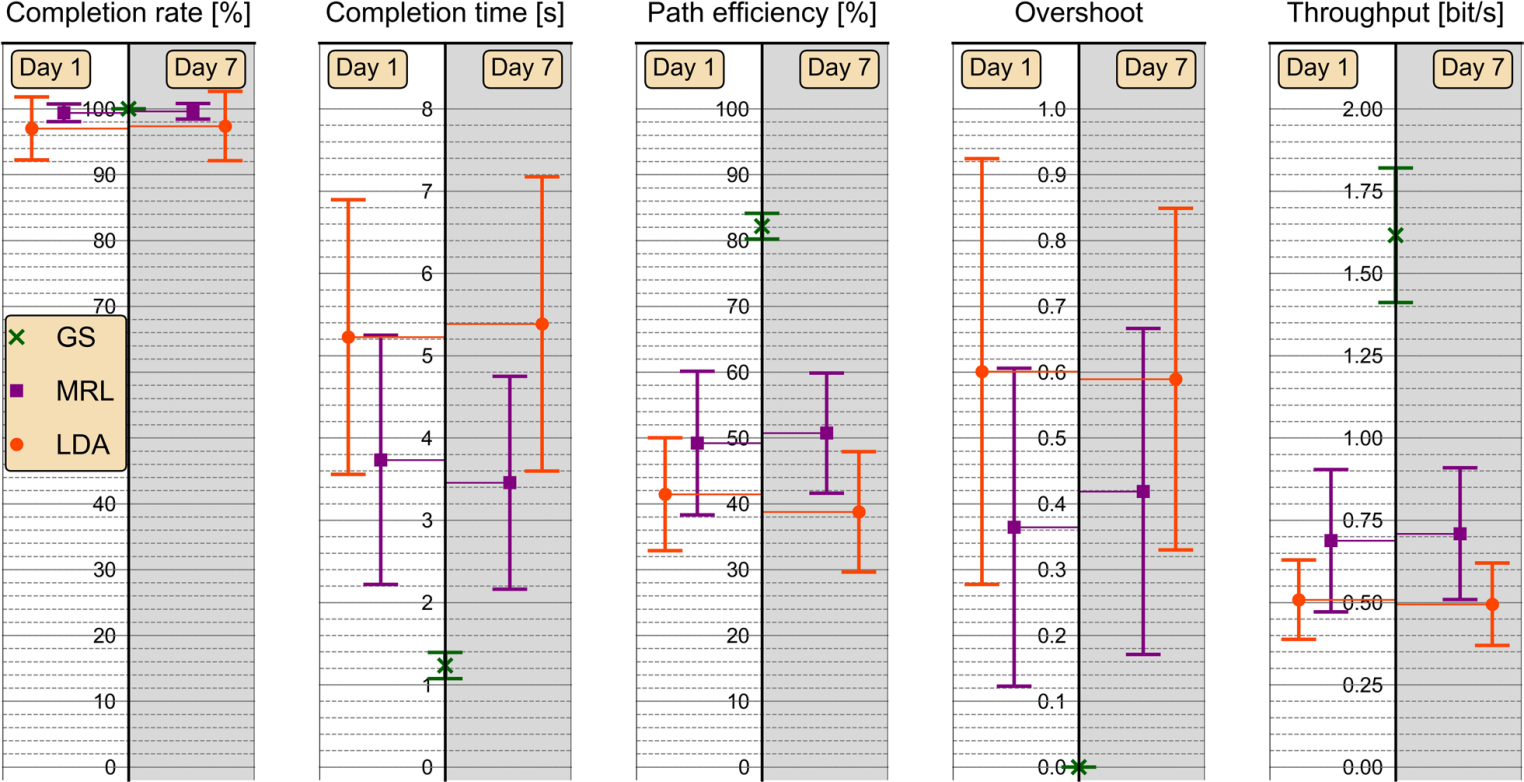
Graphical summary of myocontrol performance metrics. Metrics of motion-normalized proportional linear discriminant analysis (LDA) and the proposed myoelectric representation learning (MRL) method were obtained from evaluations conducted immediately following calibration (day 1) and after one week of intermission (day 7). Performances achieved by test subjects using the gold standard (GS), i.e. a computer mouse, are included for reference. Markers and error bars represent arithmetic means and standard deviations, respectively, across all 20 test subjects

Summary statistics of performance metrics obtained with all methods during day 1 and during day 7 are presented in Table 4 and in Table 5, respectively; the same data are summarized graphically in Fig. 8. MANOVA detected a significant effect of method ($p=0.012$) on the full set of 5 performance metrics. No significant effect of neither session ($p=1.00$) nor the interaction term method*session ($p=0.070$) was detected.

**Table 4:** Means and standard deviations of performance metrics obtained on day 1

Metric	Method		
	LDA	MRL	GS
$CR$[%]	97.00 ± 4.78	99.38 ± 1.34	100.0 ± 0.0
$CT$[s]	5.23 ± 1.67	3.73 ± 1.51	1.23 ± 0.16
$PE$[%]	41.42 ± 8.58	49.20 ± 10.91	82.17 ± 1.96
$O$	0.60 ± 0.32	0.36 ± 0.24	0.00 ± 0.00
$T$[bits/s]	0.51 ± 0.12	0.69 ± 0.22	1.62 ± 0.20

**Table 5:** Means and standard deviations of performance metrics obtained on day 7

Metric	Method	
	LDA	MRL
$CR$[%]	97.38 ± 5.27	99.63 ± 1.19
$CT$[s]	5.38 ± 1.79	3.45 ± 1.29
$PE$[%]	38.75 ± 9.15	50.73 ± 9.13
$O$	0.59 ± 0.26	0.42 ± 0.25
$T$[bits / s]	0.49 ± 0.13	0.71 ± 0.20

For $CR$, ANOVA detected a significant effect of method ($p=0.044$) and non-significant effects of session ($p=1.00$) and method*session ($p=1.00$). The effect size separating the mean value of CR across both sessions for MRL (99.50%) from that of LDA (97.19%) was computed as $d=0.62$.

For $CT$, ANOVA detected a significant effect of method ($p=0.000042$) and non-significant effects of session ($p=1.00$) and method*session ($p=0.62$). The effect size separating the mean value of $CT$ across both sessions for MRL (3.59 s) from that of LDA (5.31 s) was computed as $d=-1.07$.

For $PE$, ANOVA detected a significant effect of method ($p=0.00030$) and non-significant effects of session ($p=1.00$) and method*session ($p=0.27$). The effect size separating the mean value of $PE$ across both sessions for MRL (49.96%) from that of LDA (40.09%) was computed as $d=1.02$.

For $O$, ANOVA detected a significant effect of method ($p=0.016$) and non-significant effects of session ($p=1.00$) and method*session ($p=1.00$). The effect size separating the mean value of $O$ across both sessions for MRL (0.39) from that of LDA (0.60) was computed as $d=-0.74$.

For $T$, ANOVA detected a significant effect of method ($p=0.00033$) and non-significant effects of session ($p=1.00$) and method*session ($p=0.97$). The effect size separating the mean value of $T$ across both sessions for MRL (0.69 bits/s) from that of LDA (0.50 bits/s) was computed as $d=1.13$.

## Discussion

The main aim of the current study was to empirically evaluate the hypothesized advantages of using the proposed MRL framework for myoelectric control. For all considered performance metrics, a significant advantage was detected for MRL over LDA. For the $T$ metric, which characterize overall efficacy of control in terms of transmitted information, the effect size separating MRL from the benchmark LDA method was computed as $d=1.13$, showing that subjects achieved slightly more than one standard deviation higher performance with MRL than with LDA. For metrics characterizing more specific aspects of myoelectric control quality, the results show that the performance of MRL surpassed that of LDA, although sometimes to a lesser (but still consistently significant) degree. Anecdotally, all but 2 subjects independently expressed preference for MRL over LDA while still blind to the experimental condition, some noting that control with the latter sometimes resulted in unpredictable cursor movements, whereas the former allowed for ‘smoother’ steering and was conducive to ‘course-corrections’ if the cursor did not follow the initially planned trajectory.

Contrary to what was expected in light of some previous findings [33], the current study failed to detect any statistically significant deterioration in performance over time for either control method (neither MANOVA nor ANOVA detected any significant effect of session or method*session on any metric). A potential explanation for this discrepancy lies in the properties of the EMG recording setup used in the current study: as the electrodes had relatively large pickup area compared to what is typical, the signals they acquired could plausibly be resistant to small variations in electrode positioning over time [65]. Although it is also consistent with previous findings [66–68] to assume that subjects underwent continuous motor learning, which potentially obscures the effects of drift in EMG distribution, the results are nevertheless encouraging in that they indicate that MRL does not require frequent recalibration in order to retain its advantages over the LDA approach.

In addition to validating the MRL framework, the findings presented in the current study support a favourable view of myoelectric control based on regression of kinematics (i.e. proportional estimation of multiple DoFs simultaneously) more generally. As was experienced by the subjects of the current study, incorrect estimations can have a substantial effect on the perceived quality of control when the space of possible movements is quantized, as is the case with LDA. Conversely, errors on the part of the continuous MRL algorithm were perceived as easier to counteract, allegedly due to the less ‘jittery’ nature of the resulting interface. Compared to many conventional methods with this advantage, the type of regression proposed in the current study requires neither continuous target values nor mirrored training, allowing for straightforward use by both unilateral and bilateral amputees.

In a manner similar to the current study, one previous approach has applied regression models calibrated with entirely categorical target values for the task of myoelectric control of hand prostheses (see e.g. [69]). This earlier approach, termed on–off goal-directed training [70], applies ridge regression to estimate a linear mapping from EMG features to concurrent multi-label visual movement instruction stimulus. Once calibrated, the linear regression model use EMG to infer continuous force-patterns exerted by the digits in real-time. Notably, due to its assumptions, this method requires an approximately linear relationship between the selected EMG features and activation intensity to hold true for all steerable output DoFs in order to function effectively. This is not the case for the MRL framework introduced in the current study: a large class of possible nonlinear relationships between EMG and kinematics can be learned by the proposed ANN model during calibration. Consequently, the nonlinearity of the relationship between EMG and kinematics need not be fully captured by the selection of EMG features (i.e. the signal envelope in the current study) used as input to the MRL network model.

A salient limitation of the MRL framework as presented here relates to scalability with regard to the number of decodable DoFs. The current study was successful in extracting kinematics concerning two DoFs, but required calibration data of every possible movement combination, resulting in the recording of ${3}^{2}=9$ movements. As the number of performable movement combinations ${3}^{J}$ grows geometrically with the number of independent DoFs $J$, larger numbers of DoFs quickly lead to infeasible calibration data acquisition durations. This drawback is not unique to MRL but is, to the best of our knowledge, shared by all contemporary pattern recognition frameworks aimed at multiarticulate control. Future work could potentially focus on solving this problem by formulating generative sEMG models to artificially provide compound movement calibration data using subject-specific signals acquired exclusively from 1-DoF movements.

One avenue of algorithmic improvement for MRL concerns the preprocessing of sEMG signals. In the current study, the choice was made to let the ANN model operate on sEMG envelopes; this was motivated by the observed monotonic relationship between kinematics and sEMG magnitude, which is a necessary property to allow for the application of proportionality-inducing contractive regularization. However, a body of previous work (cf. [11]) has been unanimous in establishing that higher frequency content of sEMG signals reflects factors of the movement-dependent generative process, making information encoded at such frequencies discriminative for the purpose of movement decoding. With the MRL approach introduced and used in the current study, most high frequency information is discarded (due to envelope extraction) and cannot impact the estimation of kinematics. A question which could warrant further investigation is whether MRL can be extended to include more sophisticated sEMG signal features (handcrafted or learned) while at the same time guaranteeing proportionality by keeping the regression output constrained by the magnitude of the sEMG envelope.

## Conclusions

This paper has introduced an algorithm (MRL), based on regularized multitask learning, for the purpose of extracting proportionally encoded hand- and wrist movement intent pertaining to multiple DoFs from the forearm sEMG. To investigate the suitability of the proposed framework for use with muscle-computer interfaces, it was evaluated on able-bodied subjects with a Fitts’s law type test and compared to a standard approach, based on LDA, for myocontrol. MRL was found to be superior to LDA in the sense of significantly outperforming the latter in all considered metrics of real-time efficacy of control. Furthermore, neither MRL nor LDA could be demonstrated to undergo significant deterioration in any performance metric over a time of 7 days. Although these findings are promising, future work will have to examine performance over a longer time period in order to ascertain long-term stability.

In addition to its advantages related to efficacy of control, the proposed MRL system is computationally lightweight and can operate in real-time with relatively restricted hardware resources. Future work could thus focus on implementing this approach in the context of wearable computing platforms without expectations of reduced quality of control. Such endeavours should ideally study control of physically instantiated robotic limbs and involve forearm amputees.

## Data Availability

All data and code used for this study are available from the corresponding author on reasonable request.

## Abbreviations

ANN: Artificial neural network
ANOVA: Analysis of variance
CR: Completion rate
CT: Completion time
DoF: Degree of freedom
EMG: Electromyogram
GS: Gold standard
ID: Index of difficulty
LDA: Linear discriminant analysis
MANOVA: Multivariate analysis of variance
MAV: Mean absolute value
MCI: Muscle–computer interface
MRL: Myoelectric representation learning
MVC: Maximum voluntary contraction
O: Overshoot
PE: Path efficiency
T: Throughput
